# A direct interaction between two Restless Legs Syndrome predisposing genes: *MEIS1* and *SKOR1*

**DOI:** 10.1038/s41598-018-30665-6

**Published:** 2018-08-15

**Authors:** Helene Catoire, Faezeh Sarayloo, Karim Mourabit Amari, Sergio Apuzzo, Alanna Grant, Daniel Rochefort, Lan Xiong, Jacques Montplaisir, Christopher J. Earley, Gustavo Turecki, Patrick A. Dion, Guy A. Rouleau

**Affiliations:** 1 McGill University, Montreal Neurological Institute, Montréal, QC H3A 1A1 Canada; 2 McGill University, Department of Human Genetics, Montréal, QC H3A 1A1 Canada; 3 Centre Hospitalier de l’Université de Montréal Research Center, Montréal, QC H2L 2W5 Canada; 4 McGill University, Department of Neurology and Neurosurgery, Montréal, QC H3A 2B4 Canada; 5 Université de Montréal, Département de psychiatrie, Laboratoire de neurogénétique, Centre de recherche, Institut universitaire en santé mentale de Montréal, Montréal, QC H1N 3V2 Canada; 6 Johns Hopkins University, Department of Neurology, Hopkins Bayview Medical Center, Baltimore, MD 21224 USA; 7 McGill University, Department of Psychiatry, McGill Group for Suicide Studies, Douglas Institute, Montréal, QC H4H 1R3 Canada

## Abstract

Restless Legs syndrome (RLS) is a common sleep disorder for which the genetic contribution remains poorly explained. In 2007, the first large scale genome wide association study (GWAS) identified three genomic regions associated with RLS. *MEIS1*, *BTBD9 and MAP2K5*/*SKOR1* are the only known genes located within these loci and their association with RLS was subsequently confirmed in a number of follow up GWAS. Following this finding, our group reported the *MEIS1* risk haplotype to be associated with its decreased expression at the mRNA and protein levels. Here we report the effect of the risk variants of the three other genes strongly associated with RLS. While these variants had no effect on the mRNA levels of the genes harboring them, we find that the homeobox transcription factor *MEIS1* positively regulates the expression of the transcription co-repressor *SKOR1*. This regulation appears mediated through the binding of MEIS1 at two specific sites located in the *SKOR1* promoter region and is modified by an RLS associated SNP in the promoter region of the gene. Our findings directly link *MEIS1* and *SKOR1*, two significantly associated genes with RLS and also prioritize *SKOR1* over *MAP2K5* in the RLS associated intergenic region of *MAP2K5*/*SKOR1* found by GWAS.

## Introduction

Restless Legs syndrome (RLS), also known as Willis-Ekbom disease (WED) or Wittmaack-Ekbom syndrome, is a common sleep-related sensorimotor disorder characterized by an urge to move the legs to relieve uncomfortable sensations. These sensations occur, or worsen, during rest, such as before falling asleep or during the night^1^. RLS is a circadian sensorimotor dysregulation disorder that can lead to severe sleep disturbances and impaired quality of life^2,3^. Epidemiological studies have established that RLS is a common neurological disorder with a prevalence of up to 15% in Central Europe and North America^4–12^.

In 2007, a genome-wide association study (GWAS) conducted using German and French-Canadian RLS cases and control individuals identified variants in three genomic regions located on chromosomes 2p, 6p and 15q^13^. One of these loci was in an intronic region of the homeobox gene *MEIS1*. A second locus was in the *BTBD9* gene, which encodes a BTB (POZ) domain. The third locus was between the *MAP2K5* gene, which encodes a mitogen-activated protein kinase and the *SKOR1* gene, which encodes a transcription factor (in the 2007 study, *SKOR1* was previously referred to as *LBXCOR1*)^13^. During the same year, an independent GWAS studying cases and controls from Iceland also identified an association between *BTBD9* and periodic limb movements in sleep (PLMS), a motor feature strongly associated with RLS^14^. A third independent GWAS conducted with cases from the United States also replicated the association of *MEIS1* and *BTBD9* with RLS^15^. Two years later our group further confirmed the association between *MEIS1* and RLS as we established that the intronic risk haplotype (rs12469063/rs2300478: GG/GG) led to decreased expression of the gene at both the mRNA and protein levels in both brain tissues and lymphoblastoid cell lines (LCL) derived from patients^16^.

The first line of treatment for RLS are dopamine agonists like levodopa, suggesting that reduced dopaminergic activity is involved in the pathogenesis^17^. However, there can be negative side effects to the administration of dopaminergic drugs, like augmentation of overall RLS symptoms^18^. Iron deficiency anemia, end stage renal disease and multiple pregnancies increase the risk of RLS. The fact that iron supplements relieve symptoms in many of these cases strongly implicated iron in disease pathogenesis^19^. Studies suggest a dysfunction in the transfer of iron between the serum and the central nervous system, possibly involving the blood brain barrier^20–22^.

To extend on these previous reports and explore the pathogenic molecular mechanisms we looked for interactions between the different RLS-associated genes. First, we investigated the effects of the risk variants on the mRNA expression levels of the genes harboring them and the other RLS associated genes (*MEIS1*, *BTBD9*, *MAP2K5* and *SKOR1*). The positive interaction between *MEIS1* and *SKOR1* was further explored using specific protein-DNA binding studies and promoter reporter assays.

## Results

### *BTBD9*, *MAP2K5* and *SKOR1* expression in LCL, thalamus and pons samples of RLS patients with the corresponding risk variants

Given our previous report highlighting the decreased levels of *MEIS1* mRNA and protein in LCL and thalamus in RLS cases who carry a *MEIS1*-risk haplotype^16^, we examined if other RLS GWAS-risk variants^13,15,23^ might also affect the expression of the genes harboring them. For this purpose we used quantitative RT-PCR Taqman assays and biological material (LCL and brain tissues: pons and thalamus) to test for variations in the expression levels of these genes in RLS patients with these risk variants, similar to our previous report^16^.

**Figure 1:**
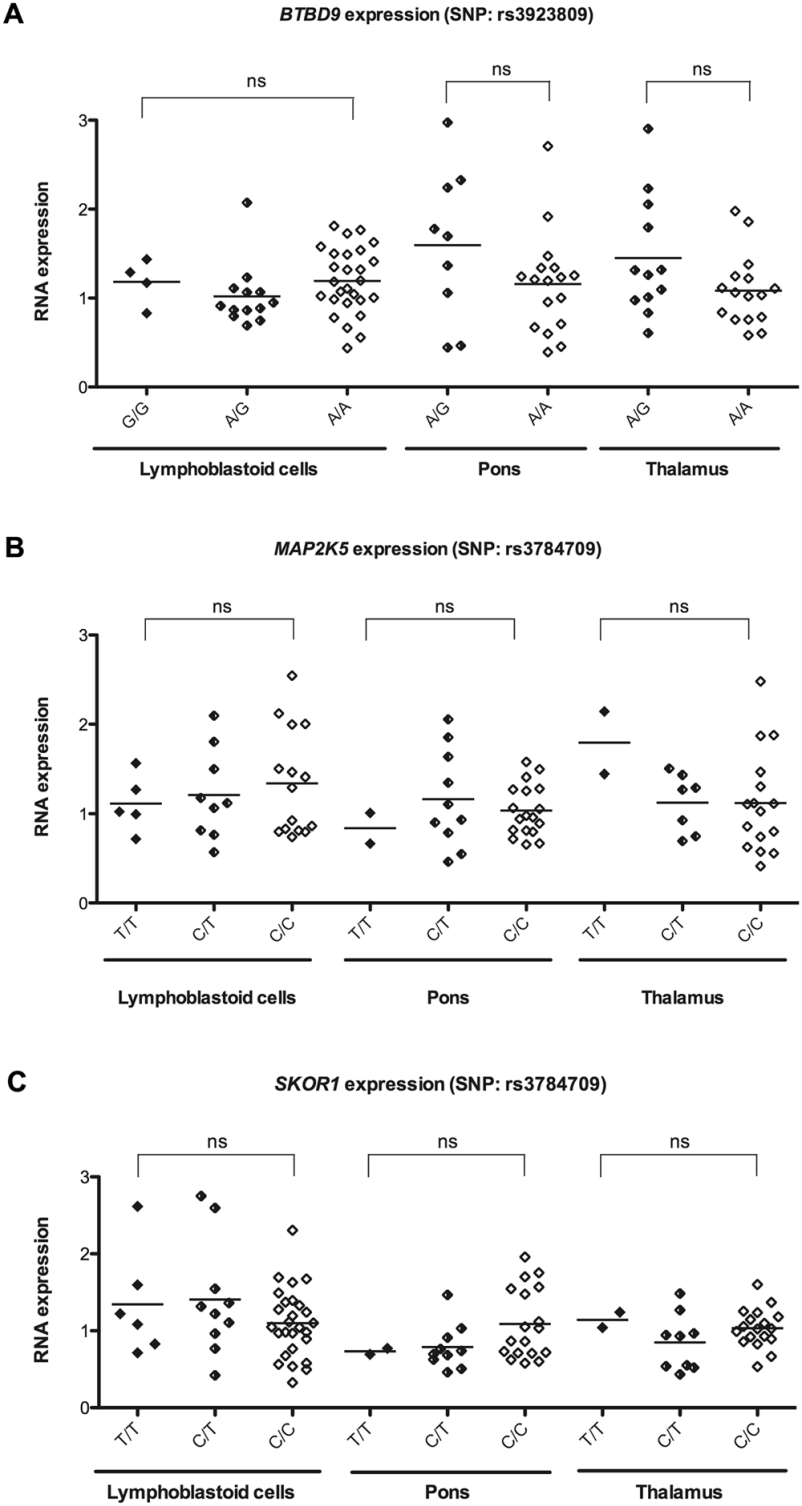
**(A)** *BTBD9* expression as a function of the genotype of the SNP rs3923809 identified in the GWAS (common allele A as the risk allele) in 44 cases of LCL, 26 cases of pons and 28 cases of thalamus. *BTBD9* expression was measured using quantitative RT-PCR Taqman method and did not show any significant difference in LCL or brains from RLS patients. **(B)** *MAP2K5* expression as a function of the genotype of the SNP rs3784709 identified in the GWAS (common allele C as the risk allele) in 29 cases of LCL, 30 cases of pons and 25 cases of thalamus. *MAP2K5* expression was measured using the quantitative RT-PCR Taqman method and did not show any significant difference in LCL or brains from RLS patients. **(C)** *SKOR1* expression as a function of the genotype of the same SNP rs3784709 identified in the GWAS (C as the risk allele) in 43 cases of LCL, 30 cases of pons and 29 cases of thalamus. *SKOR1* expression was measured using the quantitative RT-PCR Taqman and did not reveal any significant difference in LCL or brains from RLS patients.

First, we tested the rs3923809 SNP (A as the risk allele) that is located in intron 5 of *BTBD9* (Fig. 1A). When mRNA expression was measured in LCL no significant variations were seen between individuals with G/G, A/G and A/A alleles, though we had access to only four LCL of individuals homozygous for the non-risk allele (G/G). Next we measured mRNA levels of two brain regions to further explore the effects of this SNP. On average in the pons and thalamus of RLS patients who were heterozygous (A/G) carriers of the risk allele there appeared to be an increased level of *BTBD9* mRNA, though the distribution of individual samples was too broad to conclude if there was a significant difference between individuals carrying either the A/A or A/G alleles. Moreover, the lack of access to brains from cases homozygous for the G/G allele prevented us from arriving at a conclusion.

Next we tested the rs3784709 SNP (C as the risk allele) located in the intergenic region between *MAP2K5* and *SKOR1*. No effect was observed to be driven by this risk allele (which is the common allele) on the mRNA expression of either *MAP2K5* (Fig. 1B) or *SKOR1* (Fig. 1C); unfortunately the number of RLS brain samples from cases that were homozygous for the rare allele was limited. Nonetheless, the quantitative RT-PCR Taqman assays prepared from LCL suggest the mRNA levels of the two genes were not affected by the presence of the risk allele.

### *SKOR1* expression is linked to the *MEIS1* risk haplotype

**Figure 2:**
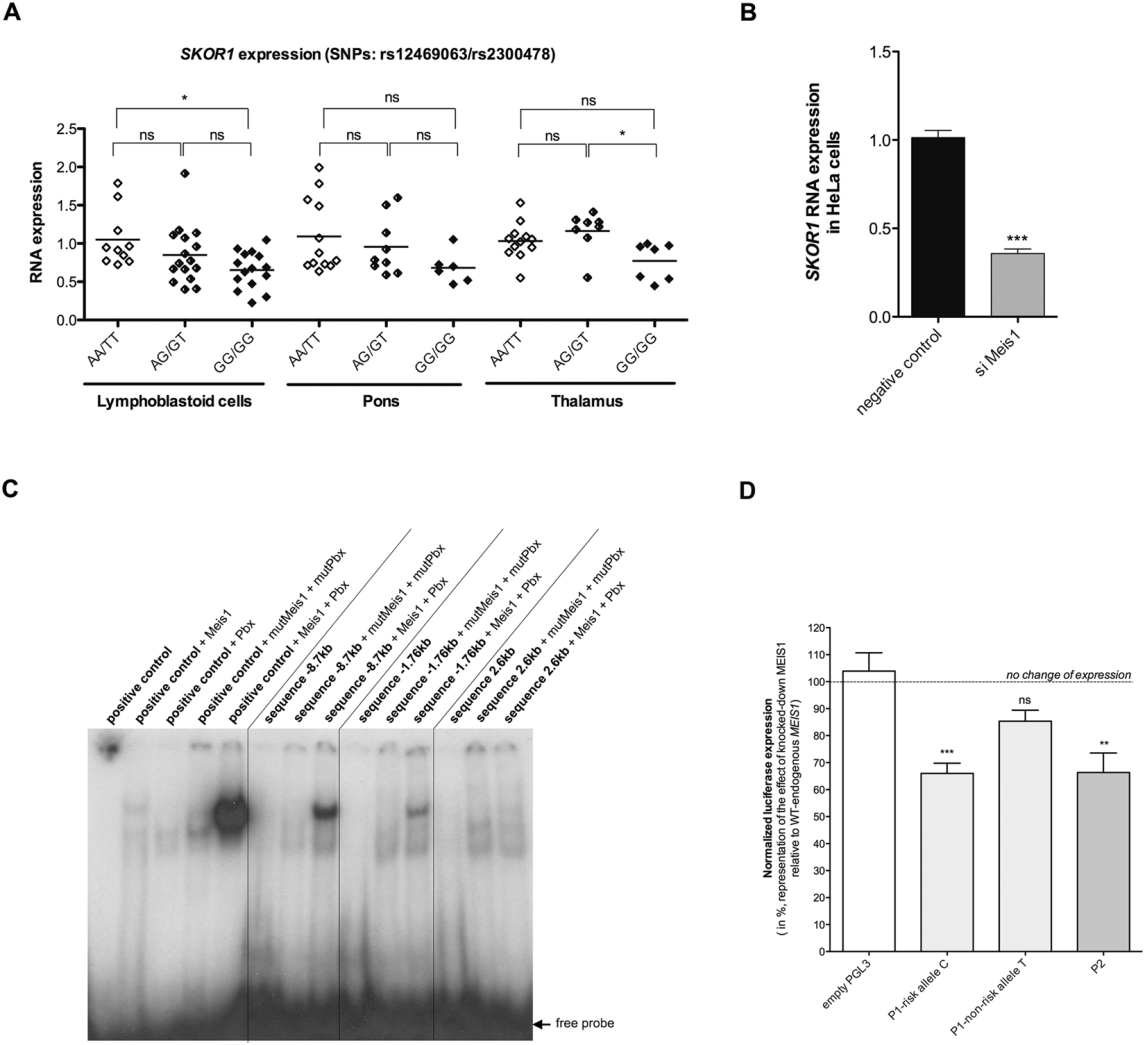
**(A)** *SKOR1* expression as a function of *MEIS1* genotype in 41 cases of LCL, 27 cases of pons and 27 cases of thalamus. *SKOR1* expression measured using the quantitative RT-PCR Taqman method showed a significant decrease of expression in LCL and thalamus with the *MEIS1* risk haplotype (GG/GG). **(B)** *SKOR1* expression in human HeLa cells after silencing *MEIS1* gene by siRNA (70% decreased expression). **(C)** Electrophoretic mobility shift assay showing the binding of MEIS1 and PBX1 on the positive control DNA portion only when together and on the three different DNA portions containing the three potential binding sites. (see Supplemental data, Fig. S4 for original image) **(D)** Luciferase assay. The results are calculated as a ratio of luciferase expression (ratio firefly / Renilla luciferase expression) and are presented as the percentage of samples with knocked-down *MEIS1* expression (treated with siRNA directed against *MEIS1*) in comparison to samples with wild-type endogenous *MEIS1* expression (negative control siRNA). As shown in the figure, P1 segment with the C allele and P2 segment (no polymorphisms present) show decreased luciferase expression as a result of reduced *MEIS1* expression using siRNA. *p < 0.05, **p < 0.01 and ***p < 0.001 compared as indicated.

After establishing the effect of each risk variant on the expression level of the genes harboring them, we looked for links between each of the risk variants and the other susceptibility genes. No significant effect on expression was observed between these genes except for the case of *SKOR1* expression, which varied according to the *MEIS1* risk haplotype. Using LCL we observed a significantly decreased expression (*P* = *0*.*0202*) of *SKOR1* in RLS cases who were homozygous carriers of the *MEIS1* risk haplotype (rs12469063/rs2300478: GG/GG) by comparison to individuals who were homozygous for the *MEIS1* non-risk haplotype (AA/TT). The same *SKOR1* expression measures were made using thalamus samples obtained from individuals carrying the same *MEIS1* haplotypes and these confirmed a similar decrease of expression(*P* = *0*.*0174*). Analysis of the pons samples from the same individuals did not show a significant decrease of expression (*P* = *0*.*1519*) (Fig. 2A). The same profile, i.e. significant change in LCL and thalamus samples and non-significant trend in pons samples, was observed in our earlier *MEIS1* expression study^16^. We tried to confirm if the mRNA decrease translated to a decreased level of *SKOR1* protein. However, none of antisera we tested (commercially available or produced by us using SKOR1 peptides) were found to be reliable as all were prone to produce various patterns of non-specific signals.

### Reduced level of *MEIS1* is directly associated with reduced *SKOR1* level

We previously reported the *MEIS1* risk haplotype (GG/GG) to be associated with decreased levels of both its mRNA and protein^16^. Hence, we decided to test if silencing *MEIS1* would result in a reduction of *SKOR1* mRNA. Using an siRNA (small interfering RNA) specifically targeting *MEIS1* we reduced the expression of the gene (~70% mRNA decrease) in HeLa cells and found a coincident 64% decrease of *SKOR1* mRNA level (*P* < *0*.*0001*) (Fig. 2B). This observation further supports the existence of a link between *MEIS1* and *SKOR1* and suggests that MEIS1 is an upstream activator of *SKOR1*.

### MEIS1 binding sites within the promoter of *SKOR1*

Since *MEIS1* is a homeobox gene that encodes a DNA binding transcription factor, we hypothesized that it might regulate the expression of *SKOR1* by binding to its promoter region. A literature search revealed that MEIS1 and members of another family of homeobox genes, PBXs (pre-B cell leukemia transcription factors like Pbx1; reviewed by Longobardi *et al*.^24^) form *in vivo* heterodimeric DNA binding complexes with each other and their DNA binding is more intense when together than when either Meis1 or Pbx1 are alone^25,26^. We searched for the presence of MEIS1/PBX1 consensus binding sites^25–27^ (Supplementary Fig. 1) across ~20 kb of human genomic sequence encompassing the ATG start codon of *SKOR1*, ~20 kb of sequence downstream of the stop codon, and all of *SKOR1* intronic sequences. Keeping in mind that *MAP2K5* gene is located 12 kb upstream *SKOR1* gene, we chose to search for the binding sites of MEIS1 in the aforementioned distance of *SKOR1* ATG start site. Three potential MEIS1/PBX1 binding sites were found: one at ~8.7 kb upstream of the ATG (TGACAGgcAGgT), a second one at ~1.76 kb upstream (TGACAGagTGAg) and a third one at ~2.6 kb downstream of the ATG, in intron 2 of the canonical isoform of *SKOR1* (GGACAGtaTGAT). Our search was focused on the hexameric consensus binding site of MEIS1. Moreover a search for the octameric consensus binding site of MEIS1 (TGATTG/TAT), as reported by Penkov *et al*., did not reveal any such sites in upstream regulatory region and the fist intron of *SKOR1* gene^28^. It is also noteworthy that an examination of Meis1 ChIP-Seq (Chromatin immunoprecipitation followed by high throughput sequencing) data made in the mouse (GEO database #GSM2188919), shows two Meis1 binding sites upstream *Skor1* gene (mm8 genome assembly) which suggest they are likely real elements involved in the activation of *Skor1* (Fig. S5)^29,30^.

Through the use of pBluescript vectors with either the coding sequence of wild-type and mutated (to serve as negative controls) murine forms of the proteins (Meis1 or Pbx1) we prepared purified proteins using *TNT Coupled Reticulocyte Lysate Systems* (Promega). The purified proteins were used in electrophoretic mobility shift assay (EMSA) experiments testing the three different human MEIS1/PBX1 binding sequences (~8.7 kb and ~1.76 kb sites upstream to the ATG and the ~2.6 kb site downstream to the ATG) (Fig. 2C). The ~2.6 kb site downstream to the ATG (the only binding site conserved in mouse, rat, cow and chimpanzee) did not bind to MEIS1 (Fig. 2C) and therefore is unlikely to directly affect the expression of *SKOR1*. However, we observed strong bindings for the two other sites (~8.7 kb and ~1.76 kb upstream to the ATG) (Fig. 2C). Overall, the *in vitro* EMSA indicates that there is a physical interaction between the MEIS1 protein and the promoter of *SKOR1* at two different positions. In light of the decreased *SKOR1* expression that was observed in RLS cases who were homozygous carriers of the *MEIS1* risk haplotype (Fig. 2A), this interaction between MEIS1 and the *SKOR1* promoter region strongly suggests a direct link between those two RLS risk genes, with MEIS1 being a regulator of *SKOR1*.

### Case control association study of an additional SNP near the ~8.7 kb MEIS1 binding site

An association study including unrelated and consecutively recruited RLS patients in Montreal, Canada (n = 401) and controls (n = 588) of European ancestry, recruited as previously described^31^. The average age at enrollment of RLS patients was 52.5 ± 14.9 years, with 38.5% men. Patients were diagnosed based on the international RLS study group (IRLSSG) criteria^32^. The average age at enrollment of the control population was 53.0 ± 16.1 years, with 38.2% men. There is no significant difference in sex and age between the cases and controls. The frequency of the risk allele (C) of rs4776976 is 0.848 in RLS cases and 0.776 in controls, respectively; and the genotype distribution fits with the Hardy-Weinberg equilibrium. The p value for the association test of the C risk allele is 0.0001267. The RLS risk allele (C) of rs4776976 is the most common allele in the European population (0.8) while the non-risk T allele of rs4776976 is the less frequent (0.2); as observed on gnom Aggregation Database^33^.

We first tested if the rs4776976 SNP might influence *SKOR1* expression and so our mRNA expression data was revisited using the two possible genotypes (C and T) (Supplementary Fig. 2). No significant effect of the risk allele was observed on *SKOR1* expression in the LCL, pons or thalamus patient samples.

### Contribution of MEIS1 binding sites upstream *SKOR1*

To test how *MEIS1* influences the expression of *SKOR1* and measure to what extent the C or T alleles at rs4776976 might affect the binding of MEIS1 at the ~8.7 kb site we used dual-luciferase assay experiments to obtain quantitative measures of expression. The ~20 kb of *SKOR1* promoter region is too large to be cloned as a single fragment upstream of a firefly luciferase reporter (PGL3, Promega) and so it was divided into two separate fragments (P1 contained the ~8.7 kb binding site and nearby rs4776976 SNP (C or T) and P2 the ~1.76 kb (Supplementary Fig. 3). These two PGL3 vectors and an empty PGL3 vector were separately co-transfected into HeLa cells alongside a vector expressing a *Renilla* luciferase. To quantify the contribution of *MEIS1* on *SKOR1* expression, the different pGL3 luciferase vectors were independently co-transfected in HeLa cells with either siRNA targeting *MEIS1* or control low GC duplex siRNA to mimic the decreased expression of *MEIS1* known to be associated with its risk haplotype. For each PGL3 luciferase vector a percentage was calculated between cells co-transfected with the *MEIS1* siRNA and the negative siRNA control (the luciferase values used to calculate these ratios were normalized from the firefly and Renilla signals) (Fig. 2D).

In the presence of *MEIS1* siRNA, a significant decrease (∼40%) of luciferase expression occurred when driven by the P1 fragment with the rs4776976 C risk allele (*P* < *0*.*001*), by comparison the P1 fragment with the T rs4776976 allele does not affect the luciferase expression. This suggests that the presence of C allele that represents the common RLS risk allele results in binding of MEIS1/PBX1 to the P1 segment of *SKOR1* promoter region. When the luciferase expression was driven by the P2 fragment a significant decrease (*P* < *0*.*01*) also occurred, thus confirming this potential MEIS1/PBX1 binding site was recognized by MEIS1 in our assay. This experiment demonstrates that MEIS1 physically acts as a direct activator of *SKOR1* and shows that the rs4776976 SNP plays an important role for the binding of MEIS1 on *SKOR1* promoter, where the presence of C allele results in *SKOR1* gene being regulated by MEIS1.

## Discussion

It is important to understand if genes identified using GWA studies interact with each other, how they exert their biological effects and which are the variants driving the association^34^. We previously showed that the RLS associated risk haplotype within the *MEIS1* non-coding regions is associated with a decreased expression of this gene in the RLS patient LCL and thalamus samples^16^. Here we show that one of the consequences of this reduced expression is reduced expression of *SKOR1*. No other risk variants in the other GWAS RLS susceptibility loci show any significant correlation with the expression levels of the genes harboring them. Considering that *MEIS1*, a homeobox gene, is a transcription factor with precise temporal and spatial gene expression during development^35^, this link could be a result of transcriptional regulatory function of MEIS1 on *SKOR1*, which we confirmed using EMSA and a luciferase reporter assay.

We also report a new SNP in the ∼8.7 kb upstream region of the *SKOR1* ATG start site that is a regulatory SNP (rSNP), as it affects the MEIS1 regulation of *SKOR1*. We found that when the risk allele is present, reduced MEIS1 expression leads to reduced *SKOR1* expression, whereas with the non-risk allele there are no changes in the expression of *SKOR1* (null allele). These data suggest that a reduced level *SKOR1* expression might be relevant to the development of a subset of RLS cases, and therefore it will be important to understand what are the downstream effects of reduced SKOR1 expression. Finally, these data also suggest that the variant driving the association with RLS in the SKOR1 locus is rs4776976.

Our findings directly linking MEIS1 and *SKOR1* strongly suggest that for the GWAS locus *MAP2K5*/*SKOR1*, the gene associated with RLS is *SKOR1* and not *MAP2K5*. *SKOR1* or SKI family transcriptional corepressor 1 is highly expressed in the central nervous system of human and mouse. In human, it appears to become more restricted to Purkinje cells of the cerebellum during adulthood^36^. The murine Skor1 interacts with a homeodomain transcription factor (Lbx1) to cooperatively repress transcription selectively in dorsal horn interneurons of the developing spinal cord^37^. It also appears to be a transcriptional corepressor which among others can regulate cell fate determination in murine dorsal spinal cord^37^. The necessity of Lbx1/Skor1 for generation of GABAergic phenotypes in dorsal horn interneurons of spinal cord potentially implicate them in the modulation of pain and sensory input processing of RLS^38–40^.

In a post GWAS era, one of the initial strategies to progress from the tag SNPs to the mechanisms underlying the disorders is to use expression studies on human patient cells harboring risk variants. In this study, establishing a link between the risk haplotype within the *MEIS1* genomic region as well the MEIS1 protein as a transcription factor with the *SKOR1* promoter sequences opens an avenue for future studies on the regulatory function of MEIS1 in the RLS pathogenesis mechanism as well as emphasizing the importance of the other candidate gene, *SKOR1*. Keeping in mind *SKOR1*’s expression pattern, as well as its proposed function as a developmental co-repressor, we can proceed to the subsequent research into RLS molecular mechanisms with a greater focus on the regulatory function of MEIS1 and SKOR1, two highly significantly associated genes with RLS.

## Material and Methods

### DNA samples

Brain tissue specimens (thalamus and upper pons) were obtained from autopsy brain tissues of 31 individuals with an RLS diagnosis from the Harvard Brain Tissue Resource Center. Final diagnosis was made by an RLS expert based on available questionnaires and medical records, but blinded to the genotype information; these samples were previously used in our earlier RLS studies^16,22^.

### LCL culture

Selected LCL from RLS patients previously established by transformation with EB virus using standard protocols were grown at 37 °C and 5% CO_2_ in RPMI 1640 medium (Invitrogen) supplemented with 15% (v/v) heat-inactivated fetal calf serum (Sigma-Aldrich), 0.29 mg/ml of l-glutamine, 100 U/ml of penicillin and 100 µg/ml of streptomycin (Invitrogen). Healthy cells were harvested at an approximate density of 1 × 10^6^ cells/ml.

### RNA expression assays

Total RNA was extracted from 0.2 g of frozen brain tissue using the RNeasy^®^ Lipid Tissue kit (Qiagen) or from 5 million lymphoblastoid cells using the RNeasy^®^ kit (Qiagen). Single-stranded cDNA synthesis was performed from 1 µg of total RNA using a mix of oligo-dT and random primers and the Quantitect^®^ Reverse Transcription kit (Qiagen) or the SuperScript® VILO™ cDNA Synthesis Kit (Invitrogen). Quantitative RT-PCR was performed using the TaqMan method (Applied Biosystems) with probes and primers designed by Applied Biosystems recognizing *BTBD9* (Hs00537653_m1) and *MAP2K5* (Hs00177134_m1) and a custom probe designed at the junction of two exons of *SKOR1* (AJ1RULU). PCR conditions were as follows: 50 °C for 2 min, 95 °C for 10 min, followed by 40 cycles at 95 °C for 15 sec (denaturation) and 60 °C for 1 min (annealing and extension). Fluorescent signals were captured using the ABI PRISM^®^ 7900HT Sequence Detection System (Applied Biosystems). The level of expression was determined by converting the threshold cycle (Ct) values using the 2^−∆∆Ct^ method. Expression levels were normalized using the human 18 S ribosomal RNA (rRNA) gene with commercial primer-probe mix (Applied Biosystems) or the human polR2A control (Hs00172187_m1). All experiments were run in triplicate. Independent cDNA synthesis was carried out twice. ANOVA test and post-hoc Tukey’s test was used for statistics of all tissues with three genotypes. *t-test* was used for statistics of *BTBD9* expression in thalamus and pons with only two genotypes.

### siRNA assays

The artificial 70% decreased expression of *MEIS1* was realised by transfection of 100pmol of the *MEIS1* siRNA (*Qiagen*, *Hs_MEIS1_10*, *#SI04321331*) for 48 hours in HeLa cells using the jetPRIME® (*Polyplus*) transfection reagent. The negative control cells were transfected with low GC duplex control siRNA. Total RNA was then extracted following a classical protocol using TRIzol® (*Invitrogen*) and *SKOR1* expression measured as described above. This experiment was run in triplicate and t-test was used for statistics.

### Electrophoretic mobility shift assay

The binding of MEIS1 and Pbx1 on *SKOR1* promoter has been tested at the three potential sites with an electrophoretic mobility shift assay. Using vectors (PCS2) designed to express either wild-type or mutated (N51S for both, since 51 is a crucial position of homeobox domains) MEIS1 or Pbx1 (*provided by Dr*. *Featherstone*), purified proteins were prepared using *in vitro* transcription/translation (IVTT) (*TNT Coupled Reticulocyte Lysate Systems part #TB126*, *Promega*). On the other hand, DNA probes were prepared by annealing of the complementary following oligonucleotides (SKOR1_^+^2.6_top: tca aac ttg ggc cgg aca gta tga tta att aca gtt taa tt; SKOR1_^+^2.6_bottom: taa tta aac tgt aat taa tca tac tgt ccg gcc caa gtt tg; SKOR1_^−^1,76_top: tgc act cca gcc tgg gtg aca gag tga gtg aga ttc cat ttc; SKOR1_^−^1,76_bottom: tga aat gga atc tca ctc act ctg tca ccc agg ctg gag tgc; SKOR1_^-^8,7_top: tgg ccc cag ttc aca atg aca ggc agg tgc ctc ctc tgc ttc; SKOR1_^−^8,7_bottom: tga agc aga gga ggc acc tgc ctg tca ttg tga act ggg gcc; Consensus_top: tac tgc tgc gat gat tga cag ccg cct cg; Consensus_bottom: tcg agg cgg ctg tca atc atc gca gca gt) in SSC buffer, marked with dATP 32 P using the Large (Klenow) Fragment DNA polymerase (*Invitrogen*) and purified using Microspin™ G50 columns (*GE Healthcare*). The resulting proteins and DNA probes were incubated for 20 minutes at room temperature with Poly DiDc 0,1 U/μL (*Novagen*) and BindBuffer (Glycerol 24%, KCl 200 mM, TRIS pH 7,5 20 mM, MgCl2 6 mM, EDTA pH 8,5 0,2 mM, Dithiothreitol 2 mM) for DNA-protein interaction and the mix was then loaded on a 30% acrylamide:bisacrylamide gel which has been pre-run at 100 V for 30 minutes. Electrophoresis is performed at 125 Volts for 45 minutes. The gel is then dried under vacuum at 80 °C for 45 minutes and autoradiography is performed with a time of exposure of 48 hours.

### Constructs for luciferase assay

To perform luciferase assays, we inserted genomic fragments from the *SKOR1* promoter into PGL3 vectors expressing the firefly luciferase gene. The ~20 kb of *SKOR1* promoter region is too long for its cloning as one piece so we cut this region into two segments; each one containing one of the potential MEIS1/Pbx1 binding site. P1 is the most upstream segment from the ATG and contains the ~8.7 kb MEIS1/Pbx1 binding site. This fragment of 5,287 bp was amplified with Phusion DNA polymerase (*New England Biolabs*) from BAC clone RP11-207J8 and using the following primers: fwd_5′-gag ctc tta cgc gtg cta gcc cgg gct cga gag ggt gcc tgt ggt gtg gga cgg tag g-3′, rev_5′-ctg act aat tga gat gca gat cgc aga tct taa att gtc ttg acc cct tgc tgg ttt tt-3′. This P1 fragment was produced with 2 alternatives (P1 form with a risk allele C at the position of rs4776976 SNP and a P1 form with a T at the same position) and using QuikChange® Site-Directed Mutagenesis (*Agilent*). The resultant PCR amplicons were then cloned into the XhoI site of pGL3-promoter vector (*Promega*) containing a SV40 promoter upstream of the firefly luciferase gene with the sequence and ligation independent cloning (slic) method^41^. P2 is the closest segment to the ATG start codon and contains the ~1.76 kb MEIS1/Pbx1 binding site. This fragment of 1,535 bp was amplified from same BAC clone using the following primers: fwd_5′-gct ctt acg cgt gct agc ccg ggg tcg acg cca aaa aga ggg aag aac c-3′, rev_5′-cta att gag atg cag atc gca gat ctc gag acc agg tcc cac ttg act tg-3′. The cloning of P2 fragment was identical to P1. Resultant clones were screened for the presence of the insert and the sequence of positive clones was verified using Sanger sequencing (*Genome Quebec Innovation Centre*).

### Case Control association study

Genotyping of rs4776976 (C/T) was done using TaqMan assays C__11771023_10, following the manufacturer’s instructions. The genotypes were called using the Applied Biosystems 7900 Fast Real-Time Polymerase Chain Reaction (PCR) System and Safety Data Sheet (SDS) software (v. 2.2.2). The goodness-of-fit test with one degree of freedom (df) was applied to check for the genotype distribution deviation from the Hardy–Weinberg equilibrium. The χ^2^ and Student’s t-tests were used to determine differences in sex and age, respectively. The association test between the marker rs4776976 and RLS was performed using the PLINK program^42^.

### Luciferase assay

At day 1, 100,000 HeLa cells per well were plated in a 24-well plate. At day 2, transfection was performed with 100 ng or 200 ng of one of the following vector expressing the firefly luciferase (empty PGL3-Promoter vector; risk or non-risk variant P1, or P2 in PGL3 vectors), 100 ng of the vector containing the Renilla luciferase and 25 pmol of siRNA (*MEIS1* or negative control) using the attractene transfection reagent (*Qiagen*). After 48 hours, cells were rinsed with PBS and lysed with 100 ul of passive lysis buffer from the Dual-Luciferase® reporter assay (*Promega*). Firefly and Renilla luciferase activity was then read using the same kit and a Synergy 4 microplate reader (*Biotek*). ANOVA test was used for statistics. The luciferase expression was measured as a ratio between firefly and Renilla expression.

### Statistics

All statistical analyses of expression studies were performed using GraphPad Prism 5.0 software (GraphPad Software Inc.) by one-way ANOVA followed by post-hoc Tukey’s test for significant results. Student’s t test was only used for Fig. 1A, for thalamus and pons samples that we only had samples with two different genotypes for each and for Fig. 2B, for the comparison of the 2 transfected groups with either negative control siRNA or specific *MEIS1* siRNA. All functional assay experiments are representations of at least 3 independent experiments expressed as mean ± SEM. Statistical significance was considered with a P value less than 0.05. In the case control association study, χ2 and Student’s t-tests were used to determine differences in sex and age, respectively. The association test between the marker rs4776976 and RLS was performed using the PLINK program by basic allelic chi-square test (1df).

### Study approval

Lymphoblastoid cell lines (LCL) were prepared from individuals who signed an informed consent form before entering the study; the biobanking of LCL has been reviewed and approved by the institutional review board of McGill University (project # 2015-164, MP-CUSM-14-051). RLS brain tissues were provided by the Harvard Brain Tissue Resource Center, which is supported in part by a Public Health Service Grant (R24MH068855), with permission from the RLS Brain Bank Tissue Review Committee through the RLS Foundation. Control brain tissues were provided by the Douglas-Bell Canada Brain Bank (Douglas Mental Health University Institute, Montreal, Quebec, Canada). In the case control association study of rs4776976 with RLS, all participants signed informed consent at enrollment, and the study protocols were approved by the institutional ethics review boards. This study was approved by Comité d’éthique de la recherche du Centre hospitalier de l′Université de Montréal and McGill University ethics, all methods were performed in accordance with the relevant guidelines and regulations of McGill University (REB NEU-14-051).

## Electronic supplementary material

Supplemental Data
