# Supplementary material for: A direct interaction between two Restless Legs Syndrome predisposing genes: MEIS1 and SKOR1
Source: Sci Rep. 2018 Aug 15;8:12173. doi: 10.1038/s41598-018-30665-6 (PMC6093889; doi:10.1038/s41598-018-30665-6)
Supplement: Supplementary file 1 — Supplemental Data [file 41598_2018_30665_MOESM1_ESM.docx]

**Title:** A direct interaction between two Restless Legs Syndrome predisposing genes: *MEIS1* and *SKOR1*

**Authors:** Helene Catoire, Faezeh Sarayloo, Karim Mourabit Amari, Sergio Apuzzo, Alanna Grant, Daniel Rochefort, Lan Xiong, Jacques Montplaisir, Christopher J. Earley, Gustavo Turecki, Patrick A. Dion, Guy A. Rouleau

**Supplemental data:**


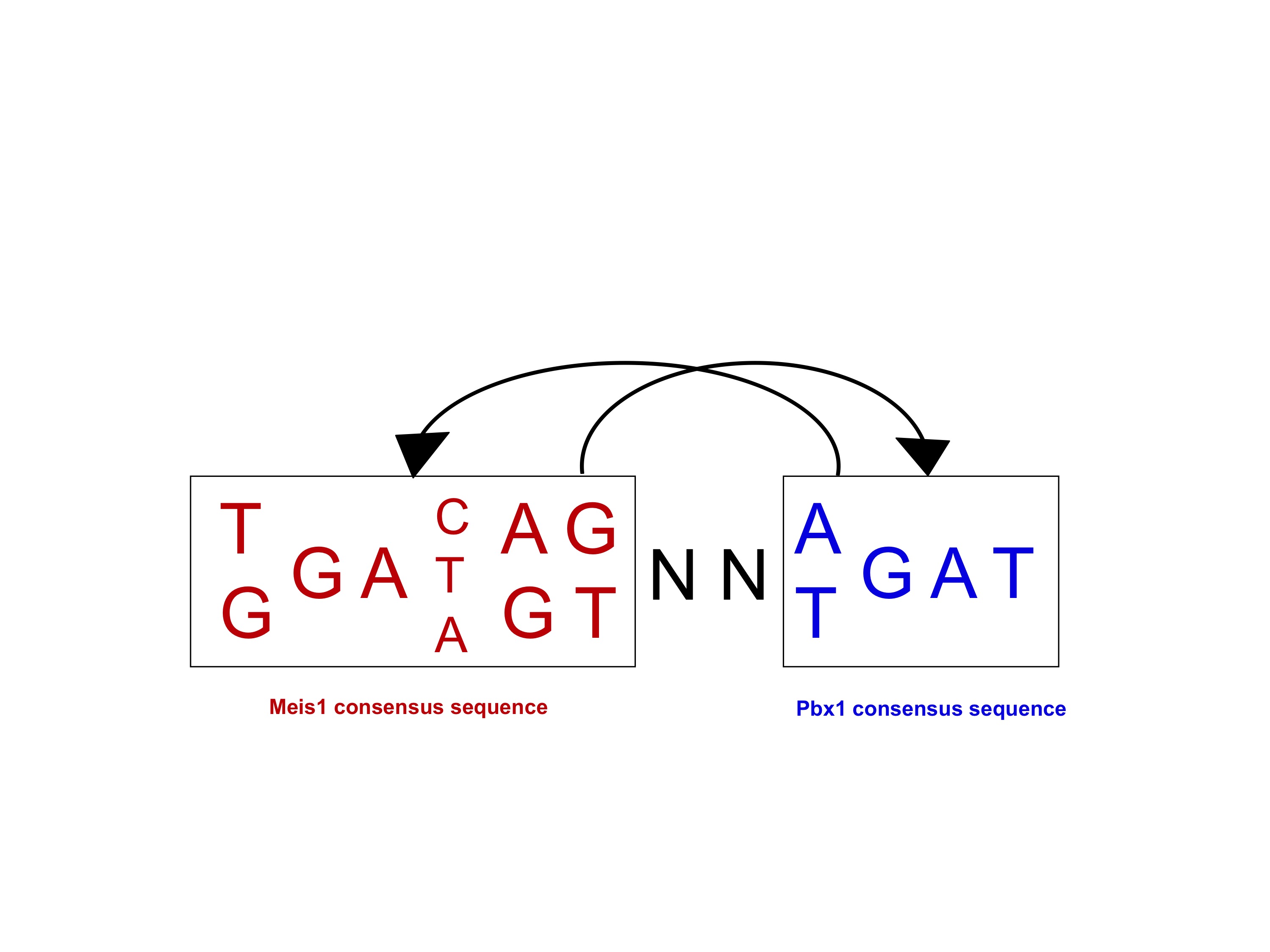


**Figure S1.** Representation of consensus sequences known to be binding sequences of MEIS1 and Pbx1.

**
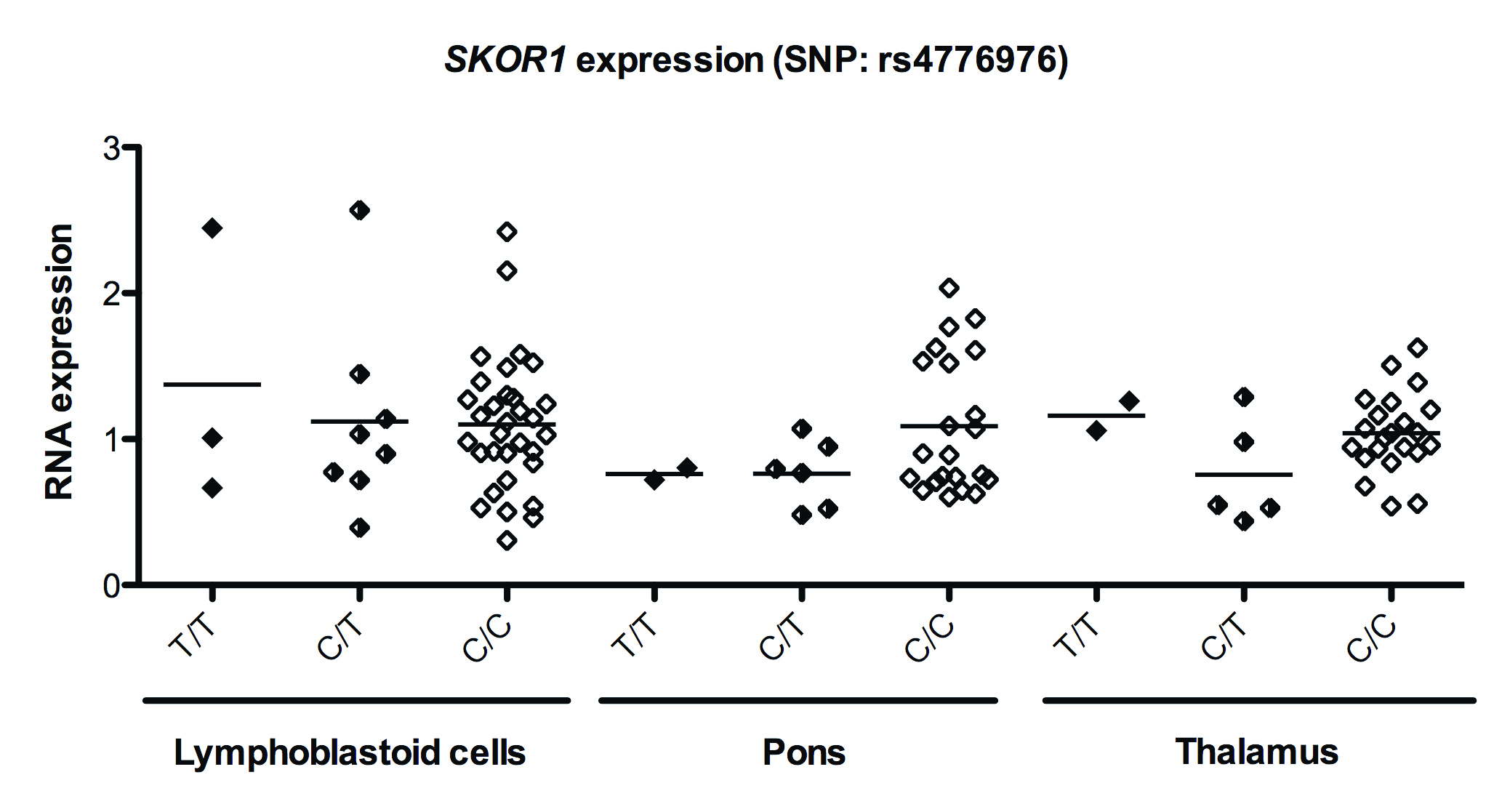
**

**Figure S2.** *SKOR1* expression as a function of the genotype of the SNP rs4776976 identified in the ~8.7kb region (common allele C as the risk allele) in 43 cases of LCL, 30 cases of pons and 29 cases of thalamus. The measure of *SKOR1* expression using quantitative RT-PCR Taqman method did not show any significant difference in LCL and brains from RLS patients.


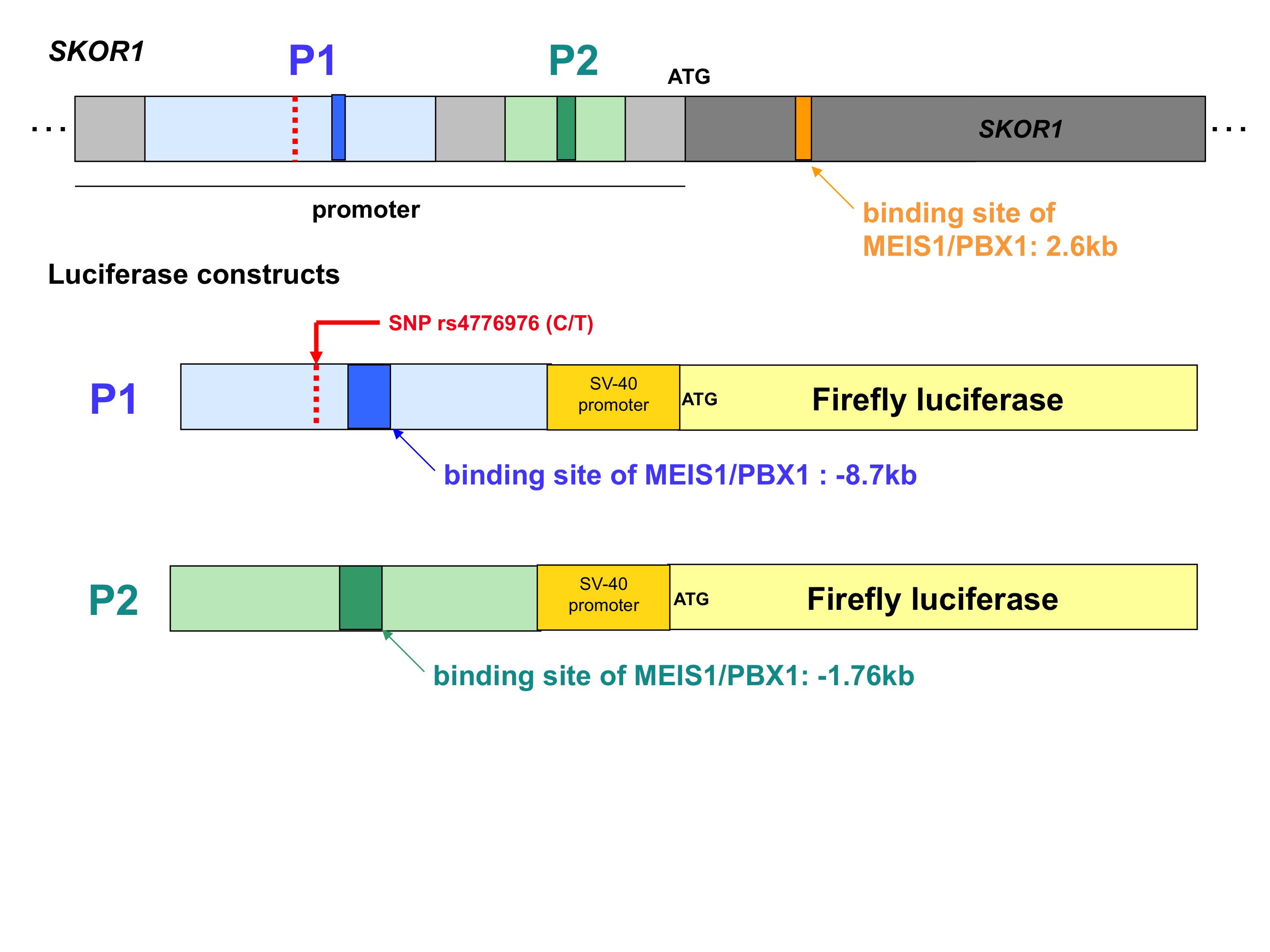


**Figure S3.** Representation of luciferase expression vectors. The *SKOR1* promoter was cut into two separate segments (P1 and P2). These fragments were inserted upstream of firefly luciferase gene in the presence of SV40 promoter to allow for sufficient expression of firefly luciferase. The SNP has been represented on the P1 fragment containing the ~8.7kb binding site of MEIS1/PBX1 and this segment was made with the two possible alleles of the SNP rs4776976, C and T.


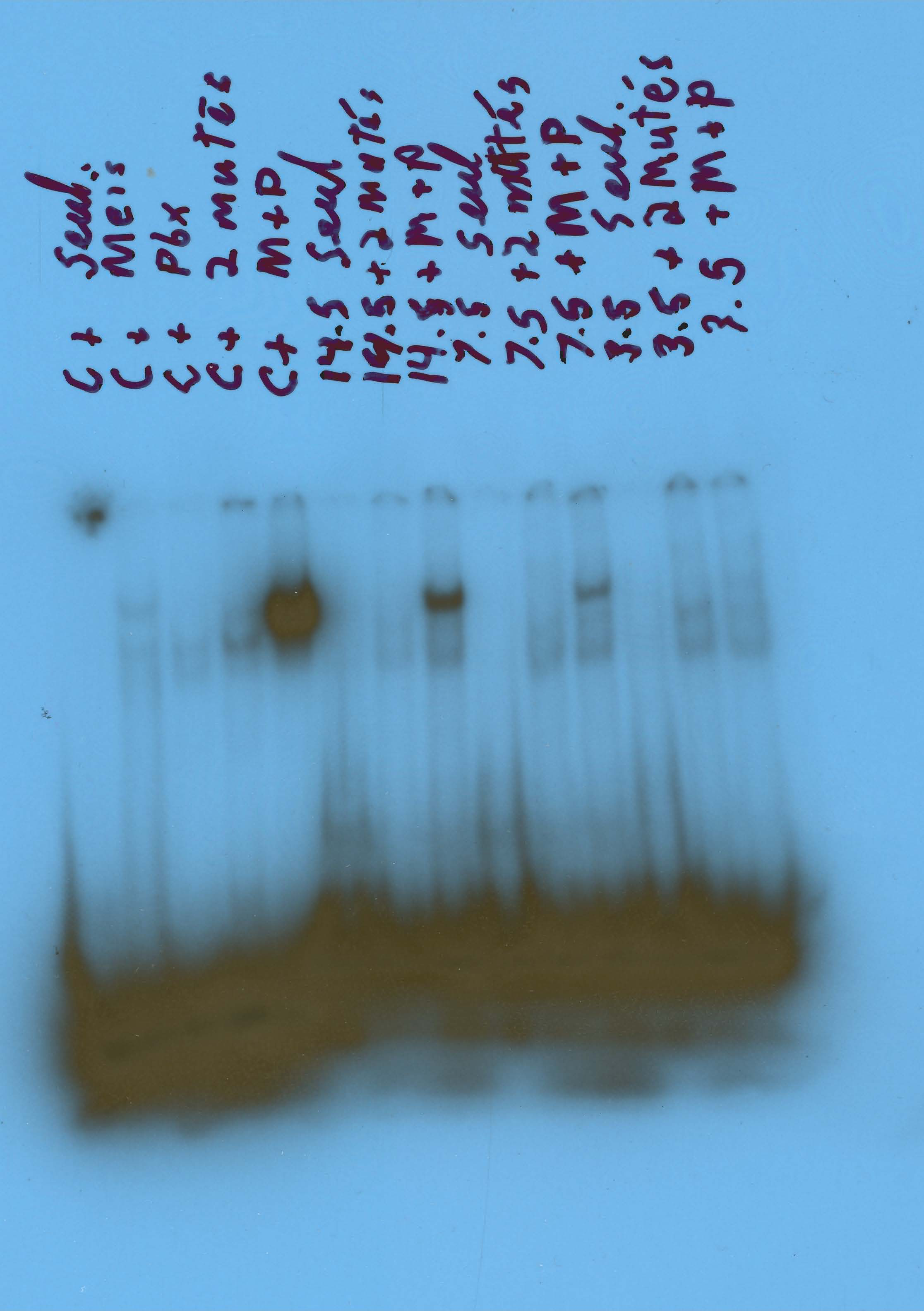


**Figure S4.** Original X-ray image corresponding to figure 2C, no touch-up, cropping or modification was done.


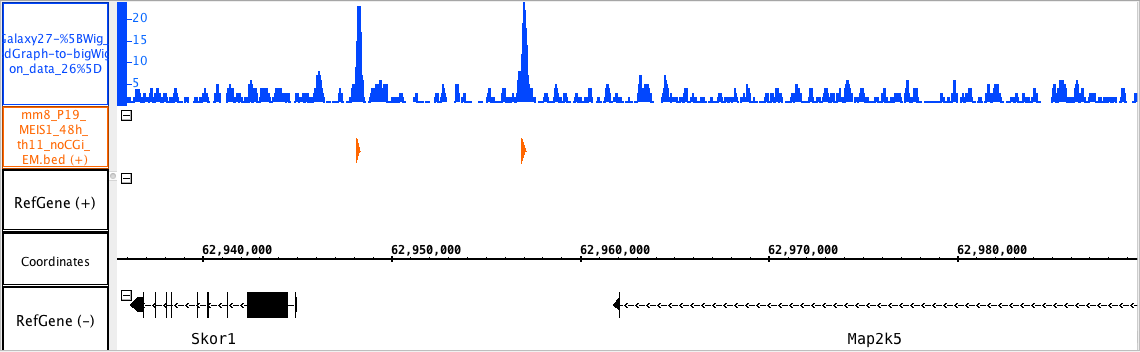


**Figure S5.** IGB view of the Meis1 ChIP-Seq data available from GEO database (GSE82314) from Mahe *et al.* Showing two peaks upstream *Skor1* gene (in orange)
